# Ginger and Its Constituents: Role in Prevention and Treatment of Gastrointestinal Cancer

**DOI:** 10.1155/2015/142979

**Published:** 2015-03-08

**Authors:** Sahdeo Prasad, Amit K. Tyagi

**Affiliations:** Cytokine Research Laboratory, Department of Experimental Therapeutics, The University of Texas MD Anderson Cancer Center, Houston, TX 77030, USA

## Abstract

Gastrointestinal (GI) cancer, a cancer of different organs of the digestive system, is one of the most common cancers around the world. The incidence and death rate of some of these cancers are very high. Although a large variety of chemotherapeutic agents have been introduced since the last few decades to combat GI cancer, most of them are very expensive and have side effects. Therefore, the compounds derived from natural sources, which are considered to be safe and cost effective, are needed. Ginger (*Zingiber officinale*) is one of the most widely used natural products consumed as a spice and medicine for treating nausea, dysentery, heartburn, flatulence, diarrhea, loss of appetite, infections, cough, and bronchitis. Experimental studies showed that ginger and its active components including 6-gingerol and 6-shogaol exert anticancer activities against GI cancer. The anticancer activity of ginger is attributed to its ability to modulate several signaling molecules like NF-*κ*B, STAT3, MAPK, PI3K, ERK1/2, Akt, TNF-*α*, COX-2, cyclin D1, cdk, MMP-9, survivin, cIAP-1, XIAP, Bcl-2, caspases, and other cell growth regulatory proteins. In this review, the evidences for the chemopreventive and chemotherapeutic potential of ginger extract and its active components using *in vitro*, animal models, and patients have been described.

## 1. Introduction

The gastrointestinal (GI) tract is one of the important parts of the body. This tract starts from the mouth, includes esophagus, stomach, small and large intestine, and rectum, and finally ends with anus. The human GI tract is a single tube which is approximately nine meters long in relaxed condition [1]. Disorder in any part of the GI tract results in various malfunctions such as diseases of digestive system and cancer.

**Figure 1:**
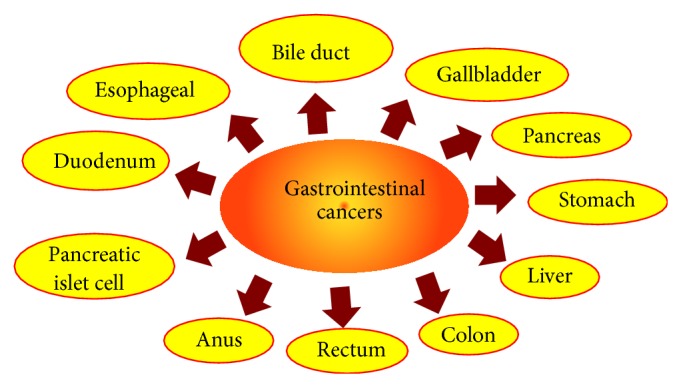
Different cancer types which are categorized under gastrointestinal cancer.

GI cancer is defined as the cancer of organs of the digestive system including the esophagus, gallbladder, liver, pancreas, stomach, small intestine, large intestine, rectum, and anus (Figure 1) [2]. The common risk factors for GI cancer include infection, smoking, drinking alcohol, high fat diet, age, race, gender, family history, and geographical location. The occurrence of GI cancer is very high in developed countries. In the United States, GI cancer accounts to 20 percent of all newly diagnosed cancer cases. Among different GI cancers, colorectal cancer is the most common cancer and is the second leading cause of death [3].

Accumulated evidences revealed that changing lifestyle could prevent all these cancers. The major change in lifestyle which proves beneficial includes avoiding tobacco, increased ingestion of fruits and vegetables, moderate use of alcohol, caloric restriction, exercise, minimal meat consumption, intake of whole grains, proper vaccinations, and regular health checkups. The link between healthy diet and cancer has been revealed in numerous studies [4–7]. An inverse association between the consumption of fruits and vegetables and cancer risk was evident by an epidemiological study in Netherlands. It has been found that the consumption of 21 vegetables and 9 fruits decreased the tumor growth in urothelial cancer patients [8]. It has also been reported that Asians have lower incidence of cancer than the residents of Western countries, and the rate increases substantially among Asians who have migrated to the West (http://www.dietandcancerreport.org/?p=ER). Consumption of diet rich in plant products may be one of the important reasons for the low incidence of cancer in Asia. A wide variety of natural products containing anticancer properties have been reported in the literature. In this paper, the role of ginger and its active ingredients against GI cancer have been discussed as it has worldwide consumption as a spice.

## 2. Ginger and Its Constituents

**Figure 2:**
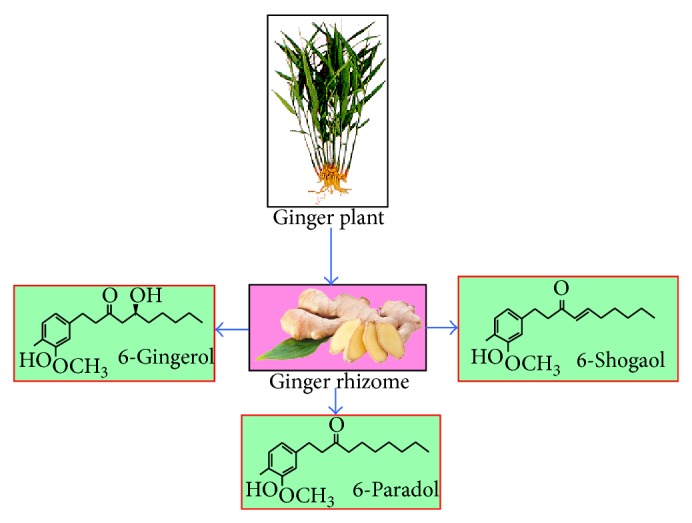
Ginger, ginger rhizome, and its major active components: 6-gingerol, 6-shogaol, and 6-paradol.

Ginger (*Zingiber officinale*), a member of the Zingiberaceae family, is a popular spice used globally especially in most of the Asian countries [9]. Chemical analysis of ginger shows that it contains over 400 different compounds. The major constituents in ginger rhizomes are carbohydrates (50–70%), lipids (3–8%), terpenes, and phenolic compounds [10]. Terpene components of ginger include zingiberene, *β*-bisabolene, *α*-farnesene, *β*-sesquiphellandrene, and *α*-curcumene, while phenolic compounds include gingerol, paradols, and shogaol (Figure 2). These gingerols (23–25%) and shogaol (18–25%) are found in higher quantity than others. Besides these, amino acids, raw fiber, ash, protein, phytosterols, vitamins (e.g., nicotinic acid and vitamin A), and minerals are also present [11, 12].

The aromatic constituents include zingiberene and bisabolene, while the pungent constituents are known as gingerols and shogaols [58]. Other gingerol- or shogaol-related compounds (1–10%), which have been reported in ginger rhizome, include 6-paradol, 1-dehydrogingerdione, 6- gingerdione and 10-gingerdione, 4- gingerdiol, 6-gingerdiol, 8-gingerdiol, and 10-gingerdiol, and diarylheptanoids [59, 60]. The characteristic odor and flavor of ginger are due to a mixture of volatile oils like shogaols and gingerols [61].

## 3. Use of Ginger as a Traditional Medicine

Ginger has been used as a spice as well as medicine in India and China since ancient times. It was also known in Europe from the 9th century and in England from the 10th century for its medicinal properties [62]. Native Americans have also used wild ginger rhizome to regulate menstruation and heartbeat. Ginger is thought to act directly on the gastrointestinal system to reduce nausea. Therefore, it is used to prevent nausea resulting from chemotherapy, motion sickness, and surgery [63]. Ginger is known as a popular remedy for nausea during pregnancy [11]. Ginger is also used to treat various types of other GI problems like morning sickness, colic, upset stomach, gas, bloating, heartburn, flatulence, diarrhea, loss of appetite, and dyspepsia (discomfort after eating). According to Indian Ayurvedic medicinal system, ginger is recommended to enhance the digestion of food [59].

Besides these, ginger has been reported as a pain relief for arthritis, muscle soreness, chest pain, low back pain, stomach pain, and menstrual pain. It can be used for treating upper respiratory tract infections, cough, and bronchitis. As an anti-inflammatory agent, it is recommended for joint problems [12]. Fresh juice of ginger has been shown to treat skin burns. Active component of ginger is used as a laxative and antacid medication. It is also used to warm the body for boosting the circulation and lowering high blood pressure. Because of its warming effect, ginger acts as antiviral for treatment of cold and flu [64]. Ginger is also used as a flavoring agent in foods and beverages and as a fragrance in soaps and cosmetics [65].

## 4. Role of Ginger and Its Constituents in Prevention and Treatment of Gastrointestinal Cancer

Evidences from* in vitro*, animal, and epidemiological studies suggest that ginger and its active constituents suppress the growth and induce apoptosis of variety of cancer types including skin, ovarian, colon, breast, cervical, oral, renal, prostate, gastric, pancreatic, liver, and brain cancer. These properties of ginger and its constituents could be associated with antioxidant, anti-inflammatory, and antimutagenic properties as well as other biological activities [66]. In this review, focus has been laid solely on GI cancers to describe whether ginger and its active components exhibit chemopreventive and chemotherapeutic potential. The* in vitro* (Table 1),* in vivo* (Table 2), and clinical effects (Table 3) of ginger have also been described.

### 4.1. Gastric Cancer

Preclinical studies have shown that ginger extract and its constituents possess chemopreventive and antineoplastic properties in gastric cancer.* In vitro* study showed that 6-gingerol induces apoptosis of gastric cancer cells. It facilitates TNF-related apoptosis-inducing ligand- (TRAIL-) induced apoptosis by increasing caspase-3/7 activation. The induction of apoptosis by 6-gingerol was mediated through downregulation of cytosolic inhibitor of apoptosis (cIAP)-1 and inhibiting TRAIL-induced nuclear factor-kappaB (NF-*κ*B) activation. Besides 6-gingerol, 6-shogaol also reduced the viability of gastric cancer cells by damaging microtubules [30]. When ginger extract was given to Sprague-Dawley rats having acetic acid-induced ulcers, it significantly reduced the gastric ulcer area. Ginger extract also attenuated elevated activities of xanthine oxidase and myeloperoxidase, as well as malondialdehyde (MDA) level in the ulcerated mucosa. Thus, ginger extract promotes ulcer healing by acting as an antioxidant and prevents gastric mucosal damage [46].

It is also reported to be effective in ameliorating the side effects of conventional therapeutic agents including *γ*-radiation, doxorubicin, and cisplatin by regulating P-glycoprotein [67]. Thus ginger extract exhibits chemosensitizing effects in certain neoplastic cells* in vitro* and* in vivo*. In support of this, another study showed that ginger reverses cisplatin-induced delay in gastric emptying indicating that ginger acts as an antiemetic for cancer chemotherapy [47]. Thus, it may be useful in improving the gastrointestinal side effects of cancer chemotherapy. Besides ginger, zerumbone (a sesquiterpene) derived from a subtropical ginger* Zingiber zerumbet* Smith was also reported to have antitumor and anti-inflammatory properties in different cancers. In gastric cancer cell lines, zerumbone inhibited cell proliferation, VEGF expression, and NF-*κ*B activation [28]. Thus, zerumbone acts as an antiangiogenic and antitumor drug in the treatment of gastric cancer.

### 4.2. Pancreatic Cancer

Ginger and its constituents are also effective against pancreatic cancer. Park et al. [27] have shown that 6-gingerol inhibits the growth of pancreatic cancer HPAC and BxPC-3 cells through cell cycle arrest at G1 phase and independent of p53 status. Further they found that 6-gingerol decreased both cyclin A and cyclin-dependent kinase (Cdk) expression followed by reduction in retinoblastoma (Rb) phosphorylation and blocking of S phase entry [27]. Another study showed that 6-gingerol regulates tight junction-related proteins and suppresses invasion and metastasis of pancreatic cancer cells. These functions of 6-gingerol were mediated through NF-*κ*B/Snail inhibition via inhibition of the extracellular signal-regulated kinases (ERK) pathway. Thus, 6-gingerol suppresses the invasive activity of PANC-1 cells [25]. Another component of ginger, 6-shogaol, triggers Ca^2+^ signals in the pancreatic *β*-cells by activating the TRPV1 channels. In fura-2 loaded single rat insulinoma (INS-1E) cells, 6-shogaol increased intracellular Ca^2+^ in a concentration-dependent manner. Intracellular Ca^2+^ increase obtained by 1 *μ*M 6-shogaol was found to be greater than that obtained by 10 mM glucose [24].

Along with* in vitro* studies, animal studies showed that 6-shogaol suppressed growth of pancreatic cancer and potentiated the effects of gemcitabine in suppression of tumor growth. The antiproliferation and sensitization to gemcitabine by 6-shogaol was mediated through the suppression of NF-*κ*B, cyclooxygenase- (COX-) 2, cyclin D1, survivin, cIAP-1, X-linked inhibitor of apoptosis protein (XIAP), Bcl-2, and matrix metallopeptidase- (MMP-) 9. It also inhibited tumor growth in pancreatic cancer xenograft model. The inhibition of this tumor growth by 6-shogaol was associated with decrease in proliferation index (Ki-67) and increased apoptosis [23]. Thus, ginger component 6-shogaol exhibits antitumor activity both* in vitro* and* in vivo*.

A component of Asian ginger, zerumbone, also inhibits growth and proliferation of pancreatic cancer through different mechanisms. It has been reported that zerumbone induces apoptosis of PANC-1 cells. The induction of apoptosis was associated with upregulation of p53 and p21 proteins as well as production of reactive oxygen species (ROS) in zerumbone-treated PANC-1 cells [26]. This result indicated that zerumbone induced apoptosis of PANC-1 cells through p53 signaling pathway. Further Sung et al. [68] showed that it inhibits invasion of pancreatic tumor cells by downregulating chemokine receptor CXCR4 expression. They also showed that the zerumbone-induced downregulation of CXCR4 was due to transcriptional regulation and inhibition of NF-*κ*B activation [68]. In support of this study, recently Shamoto et al. [22] showed that zerumbone blocks angiogenesis of pancreatic cancer cells through the inhibition of NF-*κ*B and NF-*κ*B-dependent proangiogenic gene products.

### 4.3. Liver Cancer

*In vitro* studies reveal that ginger components are effective against liver cancer. In a study, 6-shogaol has been reported to induce apoptotic cell death of Mahlavu hepatoma cells via an oxidative stress-mediated caspase-dependent mechanism. Glutathione (GSH) depletion has been shown to be a major contributing factor in arbitrating 6-shogaol-induced apoptosis of Mahlavu cells [20]. Recently Jeena et al. [40] showed that oral administration of ginger oil for one month increases antioxidant enzymes SOD, GSH, and glutathione reductase in blood of mice and glutathione-S-transferase, glutathione peroxidase, and SOD enzymes in liver of mice. Ginger oil also produced significant reduction in acute inflammation produced by carrageenan and dextran and formalin induced chronic inflammation [40], indicating its role in prevention of liver carcinogenesis.

Besides glutathione, ROS also have been involved in ginger extract-induced apoptosis of HepG2 hepatoma cells. Ginger extract at a dose of 250 *μ*g/mL markedly changes morphology of cells including cell shrinkage and condensation of chromosomes in HepG2 cells [19]. Another study showed that 6-gingerol induced apoptosis of human HepG2 cells through lysosomal-mitochondrial axis, where cathepsin D played a crucial role in the process of apoptosis. 6-Gingerol-induced release of cathepsin D preceded ROS generation and cytochrome c release from mitochondria [16]. It is also reported to protect the lipid peroxidation in liver tissue homogenate/mitochondria. The protective mechanism can be correlated to the radical scavenging property of ginger extract [69]. In animal model, ginger suppresses ethionine-induced liver carcinogenesis by scavenging the free radical formation and by reducing lipid peroxidation. Thus, ginger prevents rat hepatocarcinogenesis [44].

The major components of ginger, 6-shogaol and 6-gingerol, have shown to exert anti-invasive activity against hepatoma cells. Both compounds inhibited the migratory and invasive abilities of phorbol 12-myristate 13-acetate- (PMA-) treated HepG2 and PMA-untreated Hep3B cells. Further it was observed that inhibition of migration and invasion were mediated by decreased activity of MMP-9, urokinase-type plasminogen activator (uPA) and increased expression of tissue inhibitor metalloproteinase protein- (TIMP-) 1 [18]. Weng et al. [70] further supported their observation that 6-shogaol and 6-gingerol effectively inhibit invasion and metastasis of hepatocellular carcinoma by the inhibition of MMP-2/-9 and uPA, along with the suppression of MAPK and PI3k/Akt pathways, as well as downregulation of NF-*κ*B and STAT3 activities. In animal models, Habib et al. [43] showed that ginger extract inhibits liver carcinogenesis in Wistar rat through the downregulation of elevated NF-*κ*B and TNF-*α*. Thus, ginger may act as an anticancer and anti-inflammatory agent, which could be helpful in prevention and treatment of liver cancer.

Besides these, ginger ingredients inhibit the development of diethylnitrosamine- (DEN-) induced premalignant phenotype in rat hepatocarcinogenesis. It has been found that long-term administration of ginger extract prevented the decrease in hepatic content of metallothionein and endostatin and the increase in the growth factors induced by the carcinogen in Wistar albino rats. It also restores the serum hepatic tumor markers in rat [41]. Another study showed that 6-shogaol induces apoptosis in human hepatocellular carcinoma cells through caspase activation and ER stress signaling by regulating unfolded protein response (UPR) sensor PERK and its downstream target eIF2*α*. In mouse SMMC-7721 xenograft model, 6-shogaol inhibited tumor growth by the activation of caspase-3 and inactivation of eIF2*α* [15]. Thus, PERK/eIF2*α* pathway plays an important role in 6-shogaol-mediated ER stress and antitumorigenesis. Ginger ingredients have shown to modulate cytochrome P450 enzyme. The inhibition of CYP enzymes by ginger extract was more than its active components, gingerols [32]. Thus authors highlight the importance of consuming whole foods over active constituents.

Zerumbone was also reported to induce phase II detoxification enzymes in cultured rat normal liver epithelial cell line. In addition, it induces glutathione S-transferase in RL34 cells and it exhibits antioxidant effects by inducing nuclear localization of the transcription factor, nuclear factor- (erythroid-derived 2) like 2 (Nrf2) that binds to antioxidant response element (ARE) of the phase II enzyme genes. Thus, authors concluded that zerumbone acts as a potential activator of the Nrf2/ARE-dependent detoxification pathway that provides a new insight into cancer prevention [21]. Zerumbone has also shown to exert antitumorigenic effect in rat liver induced by DEN and 2-acetylaminofluorene. This antihepatocarcinogenic effect of zerumbone was found to be associated with suppression of PCNA and inhibition of a number of apoptotic liver cells by increased Bax and decreased Bcl-2 protein expression [42]. Thus, zerumbone has a great potential for the treatment of liver cancers.

### 4.4. Colorectal Cancer

Anticancer activities of ginger against colorectal cancer have been well documented. Numerous* in vitro* studies showed that ginger and its active components inhibit growth and proliferation of colorectal cancer cells. In a study, 6-gingerol inhibited growth of colon cancer HCT116 cells. The suppression of tumor growth was found to be linked with the inhibition of leukotriene A4 hydrolase activity, which was further confirmed by* in silico* approach [71]. Besides these, various other mechanisms were reported to be involved in 6-gingerol-induced cell growth inhibition and apoptosis in human colorectal cancer cells. These include protein degradation as well as downregulation of cyclin D1, NAG-1 beta-catenin, PKCepsilon, and GSK-3*β* pathways [35]. Radhakrishnan et al. [72] reported that the anticancer activity of 6-gingerol could be associated with the inhibition of ERK1/2/JNK/AP-1 pathway.

Whole ginger extract also prevent the primary stage of colon carcinogenesis. Administration of ginger extract to the mice pretreated with carcinogen 1,2-dimethylhydrazine (DMH) inhibited the levels of fecal bile acids, neutral sterols, tissue cholesterol, HMG CoA reductase, free fatty acids, triglycerides, phospholipase A, and phospholipase C [48]. Thus, ginger supplementation reduced the risk of colon cancer markedly by virtue of its hypolipidemic and antioxidative effects. Ginger extract not only inhibits carcinogenesis of colorectal cancer cells but also enhances the anticancer effects of chemotherapeutic drug 5-fluorouracil. It has also shown that ginger extract synergistically increases the apoptotic efficacy of Gelam honey [33]. As* in vitro*, 6-gingerol effectively suppresses tumor growth in nude mice [71].

For improving the colon cancer therapeutic efficiency of ginger extract, a multiparticulate system (ginger extract loaded with coated alginate beads) has been designed. Preclinical evaluation against DMH-induced colon cancer in male Wistar rats showed that this bead has significantly better recession of the cancers compared to free ginger extract [45]. Cysteine-conjugated shogaols have also been reported to cause death of colon cancer cells through the activation of the mitochondrial apoptotic pathway [73]. Hexahydrocurcumin extracted from ginger was also found to be cytotoxic to colorectal cancer cells. It has been observed that treatment of SW480 colon cancer cells with hexahydrocurcumin (100 *μ*M) resulted in apoptosis [74], indicating its potential as anticancer agent. Besides ginger rhizome, exposure of ginger leaf extract exhibited reduced cell viability and induced apoptosis to human colorectal cancer HCT116, SW480, and LoVo cells. This anticancer activity of ginger leaf extract was attributed to the increased expression of ATF3 through ERK1/2 activation in human colorectal cancer cells [75]. Another compound zerumbone, a sesquiterpene from the edible ginger (*Zingiber zerumbet* Smith), has been shown to enhance the radiosensitivity of colon cancer cells. It enhanced radiation-induced DNA damage and inhibited nuclear expression of DNA repair proteins ataxia-telangiectasia mutated (ATM) and DNA-PKcs [76].

## 5. Cholangiocarcinoma

*In vitro* studies showed that ginger has a promising anticancer activity against cholangiocarcinoma. Crude ethanolic extract of ginger induces cytotoxicity and antioxidant activities in cholangiocarcinoma cells. Upregulation of MDR1 and MRP3 genes was also observed by the exposure to ginger extract [77]. Using cholangiocarcinoma (KMC-1) cell line, Thatte et al. [38] reported that ginger is capable of inducing programmed cell death. In animals, intragastric treatment of ginger increases survival time and rate of animals bearing carcinogen-induced tumors [77]. In nude mouse xenograft model bearing cholangiocarcinoma tumor, ginger extract also inhibited growth of tumor and exhibited anticarcinogenic property [37]. Thus, ginger can be considered as one of the promising chemotherapeutics agents for the treatment of cholangiocarcinoma.

## 6. Clinical Studies of Ginger against GI Cancer

Besides preclinical studies, clinical studies revealed that ginger has potential for the prevention and treatment of different GI related disorders (Table 3). Study in human subjects showed that ginger delays the nausea which is stimulated during chemotherapy. In this clinical study, patients with cancer receiving chemotherapy were given normal diet, protein drink with ginger, and additional high protein with ginger twice daily. They found that protein meals with ginger reduced and delayed nausea due to chemotherapy and reduced the use of antiemetic medications [51].

In a randomized clinical study 20 subjects at increased risk for colorectal cancer were included and the patients were given 2.0 g/days ginger or placebo for 28 days. Colon biopsies were obtained to determine the levels of prostaglandin (PGE)-2, leukotriene B4 (LTB4), 13-hydroxy-octadecadienoic acids, and 5-, 12-, and 15-hydroxyeicosatetraenoic acids. They found that although ginger did not decrease eicosanoid levels in people at increased risk for colorectal cancer, however, it was both tolerable and safe [52]. Earlier in a phase II study, Zick et al. [57] have shown no significant difference in eicosanoids level in 30 people at normal risk for colorectal cancer. However, they found a significant decrease in PGE2 and 5-hydroxyeicosatetraenoic acid (HETE) and a trend toward significant decrease in 12-HETE and 15-HETE normalized to free arachidonic acid [57]. Another study on 66 colorectal cancer patients receiving chemotherapy has shown that massage with ginger and coconut oil improved cellular immunity of these patients. They found that this aromatherapy with massage boost lymphocyte number by 11%. It also decreased fatigue, presenting symptom, pain, and stress in cancer patients [53].

In another pilot, randomized control trial with 20 patients at increased risk for colorectal cancer, ginger (2 g for 28 days) supplementation was also found to reduce the proliferation of normal-appearing colorectal epithelium and increased apoptosis and differentiation of the crypts. This beneficial effect of ginger was found to be associated with downregulation of Bax, human telomerase reverse transcriptase (hTERT), and MIB-1, while p21 and Bcl-2 expression remained relatively unchanged [55]. Ginger has been reported to have anti-inflammatory activities as observed in a study with 30 normal participants and 20 participants at increased risk for colorectal cancer. It has been observed that ginger significantly lowers COX-1 protein expression in participants at increased risk for colorectal cancer but not in the participants at normal risk. However, ginger did not alter 15- hydroxyprostaglandin dehydrogenase (PGDH) protein expression in either increased or normal-risk participants [56]. These results indicate chemopreventive potential of ginger against colorectal cancer.

## 7. Molecular Targets

**Figure 3:**
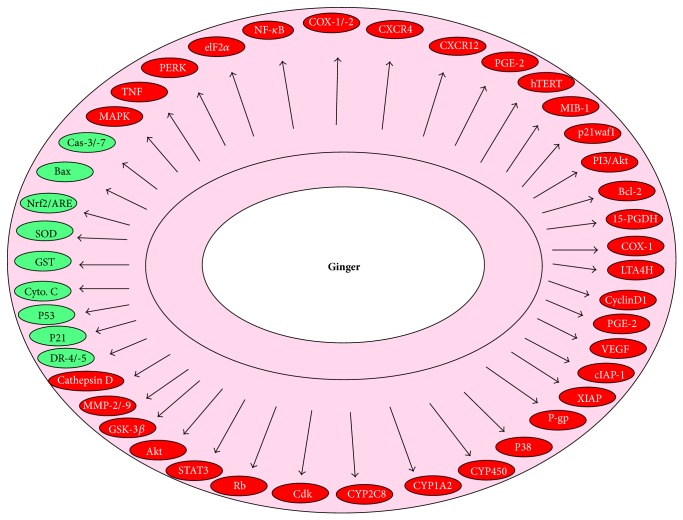
Molecular targets of ginger and its active constituents against gastrointestinal cancer.

Ginger and its components have been shown to modulate a wide range of signaling molecules (Figure 3). Ginger may upregulate or downregulate the gene expressions, depending on the target and cellular context. Ginger extract increases antioxidant enzymes including GSH, SOD, and glutathione peroxidase [40]. Component of Asian ginger oil also targets to increase the phase II detoxification enzymes as well as nuclear localization of Nrf2/ARE [21]. A number of targets of ginger and its components have been documented in different cancer models. These include transcription factors, enzymes, inflammatory mediators, protein kinases, drug resistance proteins, adhesion molecules, growth factors receptors, cell-cycle regulatory proteins, cell-survival proteins, chemokines, and chemokine receptors. In different GI cancers, ginger extract inhibits transcription factor NF-*κ*B, inflammatory cytokine TNF-*α* and other enzymes and proteins, which include xanthine oxidase and myeloperoxidase, MDA, HMG CoA reductase, free fatty acids, triglycerides, phospholipase A, and phospholipase C. The active ingredient of ginger, particularly, 6-gingerol and 6-shogaol targets several cellular molecules that contribute to tumorigenesis, cell survival, cell proliferation, invasion, and angiogenesis. 6-Gingerol modulates NF-*κ*B, STAT3, Rb, MAPK, PI3K, Akt, ERK, cIAP1, cyclin A, Cdk, cathepsin D, and caspase-3/7. Similarly, shogaol targets NF-*κ*B, STAT3, MAPK, PI3k/Akt Ca^2+^ signals, COX-2, cyclin D1, survivin, cIAP-1, XIAP, Bcl-2, MMP-9, caspase activation, ER stress, and eIF2*α*. Besides these, Asian ginger component zerumbone modulates NF-*κ*B, p53 VEGF, p21, and CXCR4 expression. Thus these molecular targets of ginger components indicate that it may have the potential for preventing and treating the GI cancer.

## 8. Conclusion

Although the medicinal properties of ginger have been known for thousands of years, a significant number of* in vitro*,* in vivo*, and epidemiological studies further provide substantial evidence that ginger and its active compounds are effective against wide variety of human diseases including GI cancer. Ginger has been found to be effective against various GI cancers such as gastric cancer, pancreatic cancer, liver cancer, colorectal cancer, and cholangiocarcinoma. However, its anticancer effects on other GI cancers like duodenal, esophageal, anal, GI carcinoid tumor and pancreatic islet cell cancer have yet not been established. Therefore, efficacy of such potent agents on these cancers is warranted. Ginger and its polyphenols have been shown to target multiple signaling molecules that provide a basis for its use against multifactorial human diseases. Moreover, most of the known activities of ginger components are based only on* in vitro* and* in vivo* studies, except for a few clinical studies in human subjects. Therefore, more extensive and well-controlled human studies are required to demonstrate its efficacy as an anticancer agent, as it is a safe and cost-effective alternative.

## Figures and Tables

**Table 1:** *In vitro* effects of ginger and its constituents against various GI cancer models.

Cancer	Effects	Reference
Liver		
HepG2	Induce apoptosis by activation of caspase-3	[13]
Liver microsomes	Inhibit CYP450, 1-aminobenzotriazole, and aldo-keto reductase Prevent the formation of M14 and M15 and 18*β*-glycyrrhetinic acid	[14]
SMMC-7721	Inhibit the phosphorylation of eIF2*α* and triggered apoptosis	[15]
HeoG2	Release cathepsin D and subsequently cytochrome c Induce apoptosis and intracellular ROS generation and reduced glutathione	[16]
PC12	Inhibit xanthine oxidase and H_2_O_2_-induced damage	[17]
HepG2/Hep3B	Decrease the MMP-9 activity and increase the TIMP-1 expression Decrease urokinase-type plasminogen activator activity in Hep3B cells	[18]
Hep-2	Dose-dependently suppress cell proliferation	[19]
Mahlavu cells	Activate caspases 3/7 resulting in the DNA fragmentation	[20]
RL34	Activate the Nrf2/ARE-dependent detoxification pathway	[21]
Pancreas		
PaCa	Inhibit mRNA expression and protein secretion of angiogenic factors and NF-*κ*B activity	[22]
PANC-1, BxPC	Downregulate of NF-*κ*B signaling and cell survival regulators including COX-2, cyclin D1, survivin, cIAP-1, XIAP, Bcl-2, and MMP-9 and sensitize to gemcitabine	[23]
*β*-cell (INS-1E)	Induce Ca^2+^ signals in the *β*-cell by activating the TRPV1 channels	[24]
PANC-1	Decrease invasion and metastasis and NF-*κ*B translocation via downregulation of the ERK pathway	[25]
PANC-1	Upregulate p53, p21 proteins level and ROS production	[26]
HPAC, BxPC-3	Decrease cyclin A, Cdk, Rb phosphorylation, and p53 expression	[27]
Gastric Cancer		
HUVE-AGS	Inhibit cell proliferation, VEGF expression, and NF-*κ*B activity	[28]
kBZ Jurkat	inhibit COX-2 activation and reduce *H. pylori*-induced inflammation	[29]
HGC/AGS/and KATO III	Inhibit TRAIL-induced NF-*κ*B activation, cIAP1 expression Increase TRAIL-induced caspase-3/7 activation	[30]
JB6	Inhibit the growth of all *Helicobacter pylori* strains	[31]
Colorectal		
Caco-2	Inhibit cytochrome P450 enzymes (CYP1A2 and CYP2C8)	[32]
HCT116	Act as antiproliferative agents and enhance the chemotherapeutic effect of 5-FU	[33]
COLO 205	Induce apoptosis, cytochrome c release, caspase activation, and DNA fragmentation Upregulate the Bax, Fas, and FasL and downregulate Bcl-2 and Bcl-XL proteins	[34]
HCT116	Suppress cyclin D1 expression and induced NAG-1 expression Inhibit beta-catenin, PKC-epsilon, and GSK-3 beta pathways	[35]
HCT116	Potentiate TRAIL-induced apoptosis and upregulate of TRAIL death receptors (DR-4/-5) Inhibit extracellular signal-regulated kinase 1/2 and p38-MAPK	[36]
Cholangiocarcinoma		
CCA (CL-6)	Upregulate MDR1 and MRP3 genes	[37]
KIM-1	Induce programmed cell death through endonuclease activation and induction of p53	[38]
KMC-1	caspase 3 activation, potentiate free-radical formation and accumulation of sphinganine	

CYP450, cytochrome P450; eIF2*α*, eukaryotic initiation factor 2 alpha; ROS, reactive oxygen species; TIMP-1, tissue inhibitor of metalloproteinase 1; Nrf2, nuclear factor (erythroid-derived 2)-like 2; ARE, antioxidant response element; COX-2, cyclooxygenase-2; cIAP-1, cellular inhibitor of apoptosis protein-1; XIAP, X-linked inhibitor of apoptosis protein; MMP-9, matrix metallopeptidase-9; NF-*κ*B, nuclear factor kappaB; ERKs, extracellular-signal-regulated kinase; Rb, retinoblastoma; VEGF, vascular endothelial growth factor; TRAIL, TNF-related apoptosis-inducing ligand; NAG-1, nonsteroidal anti-inflammatory drug- (NSAID-) activated gene-1; PKC, protein kinase C; GSK-3 beta, glycogen synthase kinase-3 beta; MDR1, multidrug resistance gene-1; MRP3; multidrug resistance protein 3.

**Table 2:** *In vivo* effects of ginger and its constituents against various GI cancer models.

Cancer	Effects	Reference
Liver	Exhibit hepatoprotective activity against alcoholic fatty liver disease in C57BL/6 mice	[39]
Liver	Increase superoxide dismutase and glutathione reductase level in blood Increase glutathione-S-transferase, glutathione peroxidase, and superoxide dismutase enzymes in liver	[40]
	Reduce carrageenan-, dextran-, and formalin- induced chronic inflammation Reduce acetic acid induced writhing movements	[41]
Liver	Decrease the hepatic content of metallothionein and endostatin in Wister Albino rats Increase the growth factors induced by the carcinogen	
Liver	Protect the rat liver from the carcinogenic effects of DEN and AAF Increase Bax and decrease Bcl-2 protein expression	[42]
	Downregulate serum alanine transaminase, aspartate transaminase, alkaline phosphatase, and alpha-fetoprotein	[23]
Pancreatic	Downregulate NF-*κ*B signaling and cell survival regulators and sensitize to gemcitabine treatment in pancreatic cancer xenografted mice	
Liver	Inhibit CYP450, 1-aminobenzotriazole, and aldo-keto reductase liver microsomes of rats and prevent the formation of M14 and M15 and 18*β*-glycyrrhetinic acid	[14]
Liver	Downregulate NF-*κ*B and TNF-*α* in Wistar rats with liver cancer	[43]
Liver	Reduce SOD activity and MDA level and increase catalase activity in liver of Wistar rats	[44]
Colon	Decrease the incidence and number of tumors in colon of Wistar rats	[45]
Gastric	Inhibit the expression of the chemokines and TNF-*α* in gastric cancer of rat model	[46]
Gastric	Reverse cisplatin-induced delay in gastric emptying in rats	[47]
Colon	Decrease the fecal bile acids, neutral sterols, tissue cholesterol, HMG CoA reductase, free fatty acids, triglycerides, phospholipase A, and phospholipase C in colon	[48]
Colon	Decrease the incidence and number of tumors in colon as well as the activity of beta-glucuronidase and mucinase	[49]
CCA	Exhibit anti-inflammatory, antihypertensive, and antiulcer activities in CCA xenograft nude mouse model	[37]
Colon	Block the azoxymethane-induced intestinal carcinogenesis in rats	[50]

DEN, diethylnitrosamine; AAF, acetylaminofluorene; NF-*κ*B, nuclear factor kappaB; CYP450, cytchrome P450; TNF-*α*, tumor necrosis factor-alpha; SOD, superoxide dismutase; MDA, malondialdehyde; CCA, cholangiocarcinoma.

**Table 3:** Beneficial effects of ginger and its constituents in GI cancer patients.

Effects	Reference
Decrease the gastric dysrhythmia and reduce the delayed nausea of chemotherapy	[51]
Inhibit COX and decrease PGE2 concentrations in colorectal cancer Decrease the incidence and multiplicity of adenomas	[52]
Increase the lymphocyte counts in colorectal cancer patients	[53]
Reduce proliferation (hTERT, MIB-1) and differentiation (p21waf1/cip1) in colon cancer	[54]
Decrease the hTERT, MIB-1, and Bax expression in the whole crypts of colon	[55]
Decrease COX-1 protein expression in participants at increased risk for colorectal cancer	[56]
Decrease the mean percent change in PGE-2 and 5-HETE levels in colorectal cancer	[57]
Inhibit CYP450, 1-aminobenzotriazole, and aldo-keto reductase in human liver microsomes	[14]
Prevent the formation of M14 and M15 and 18*β*-glycyrrhetinic acid in human liver microsomes	[14]
